# Distinct role of Sirtuin 1 (SIRT1) and Sirtuin 2 (SIRT2) in inhibiting cargo-loading and release of extracellular vesicles

**DOI:** 10.1038/s41598-019-56635-0

**Published:** 2019-12-27

**Authors:** Byung Rho Lee, Bethany J. Sanstrum, Yutao Liu, Sang-Ho Kwon

**Affiliations:** Department of Cellular Biology and Anatomy, Medical College of Georgia, Augusta University, Augusta, GA USA

**Keywords:** Secretion, Endosomes

## Abstract

Exosomes, vehicles for intercellular communication, are formed intracellularly within multivesicular bodies (MVBs) and are released upon fusion with the plasma membrane. For their biogenesis, proper cargo loading to exosomes and vesicle traffic for extracellular release are required. Previously we showed that the L-type lectin, LMAN2, limits trans-Golgi Network (TGN)-to-endosomes traffic of GPRC5B, an exosome cargo protein, for exosome release. Here, we identified that the protein deacetylase sirtuin 2 (SIRT2) as a novel interactor of LMAN2. Loss of SIRT2 expression resulted in exosomal release of LMAN2, a Golgi resident protein, along with increased exosomal release of GPRC5B. Furthermore, knockout of SIRT2 increased total number of extracellular vesicles (EVs), indicating increased MVB-to-EV flux. While knockout of SIRT1 increased EV release with enlarged late endolysosome, knockout of SIRT2 did not exhibit endolysosome enlargement for increased EV release. Taken together, our study suggests that SIRT2 regulates cargo loading to MVBs and MVB-to-EV flux through a mechanism distinct from that of SIRT1.

## Introduction

Small extracellular vesicles (EVs) called exosomes are vehicles for intercellular communication^1,2^ and are involved in the progression of many human disease or protection against diseases^3,4^. Cargoes loaded in exosomes can range from various RNAs to growth factors and extracellular remodeling molecules^3,5–7^. For the communication role, select cargo loading and proper vesicle trafficking in exosome producing cells as well as retrieval of exosomes to target cells are required. Exosomes are formed intracellularly within multivesicular bodies (MVBs) and are released when the MVB fuses with the interior of plasma membrane^1^. It has been established that endosomal sorting complexes required for transport (ESCRT) components recognize the ubiquitination of exosome cargo proteins, which can result in lysosomal degradation or alternatively can be diverted to exosome release^8^. Release of exosomes seems to require ALIX (also called PDCD6IP) which aids in the formation of an interaction between the cargo syndecan and the ESCRT-III complex suggesting that ESCRT proteins are involved in determining the fate of MVBs carrying exosomes^9^. However, the exact mechanisms of MVB fate for exosomal release are still largely undetermined.

The G-protein-coupled receptor GPRC5B is an exosome cargo protein that expresses abundantly in brain and kidney tissue^10^. We have previously shown that GPRC5B is induced during *in vitro* kidney tubule formation^11^ and exosomal release of induced GPRC5B enhances *in vitro* renal tubule growth^12^. During exosomal release of GPRC5B, the L-type lectin LMAN2 limits trans-Golgi Network (TGN)-to-endosomes traffic of GPRC5B^13^. Depletion of LMAN2 engaged GPRC5B to endosome traffic by promoting exit of GPRC5B from the TGN, suggesting LMAN2 is a negative regulator of exosomal release of GPRC5B. In light of this information, we sought to identify a novel protein interactor(s) of LMAN2 in order to delineate the role of LMAN2 in exosome biogenesis.

The sirtuin (SIRT) family of NAD-dependent deacetylases has been implicated in various human diseases such as cancer, inflammation, and aging^14,15^. The involvement of sirtuin proteins in the regulation of many health-related mechanisms makes them interesting candidates to study. SIRT1, the most studied nuclear protein, has been linked to longevity and lifespan by maintaining telomere length, caloric restriction, and age-related reactive oxygen species^16–18^. Furthermore, recent studies have reported the role of SIRT1 in exosome biogenesis^19,20^. In this study, we identified that SIRT2, another member of sirtuin protein family as an interacting protein of LMAN2 which plays a key role in exosome biogenesis. Unexpectedly, loss of SIRT2 resulted in exosomal release of LMAN2, a Golgi resident protein and increased exosomal release of GPRC5B. In addition, the total number of extracellular vesicles was increased when expression of SIRT2 was lost. Altogether, these findings suggest that the SIRT2 protein controls release of extracellular vesicles, including exosomes, at the multiple steps, including vesicle traffic of cargo proteins to exosome-destined MVB (cargo loading) and MVB-to-exosome flux (exosome biogenesis).

## Materials and Methods

### Cell culture

Isogenic Flp-In T-REx HEK293 cells inducibly expressing GPRC5B-HA^13^ were maintained in DMEM (Gibco, Cat#: 10564011) supplemented with 5% (v/v) tetracycline negative fetal bovine serum (Gemini, Cat#: 100–800), 100 units/ml penicillin (Gibco), 100 mg/ml streptomycin (Gibco), and humidified in 5% CO_2_ at 37 °C. Cells were passaged at sub-confluence and routinely checked for mycoplasma contamination, using LookOut Mycoplasma PCR detection kit (Sigma).

### Plasmid transfection and shRNA-mediated knockdown

Plasmids expressing LMAN2, SIRT, HDAC, or GFP proteins^13,21^ were transfected using Lipofectamine 2000, according to the manufacturer’s instruction.

pLKO-based Validated MISSION shRNAs (TRCN0000040221, TRCN0000040222) targeting human SIRT2 gene for human cells and negative control (SHC001) were purchased from Sigma. Plasmids carrying U6 promoter-driven shRNAs were transfected using Lipofectamine 2000, according to the manufacturer’s instruction.

### Exosome preparation

Exosome isolation was performed, as described previously^13^. Briefly, 22-to-24 hours before preparation, growth medium was replaced by serum-free medium in order to make sure the presence of all the exosome cargoes surveyed in this study were from the cell lines, not fetal bovine serum, and conditioned medium exposed to the indicated cell lines was then collected. Conditioned medium was spun at 500 × g for 20 min and at 2,000 × g for 20 min, sequentially. The supernatant was filtered through sterile 0.2 μm PES membrane. The resulting filtrate was diluted with DPBS (Dulbecco’s phosphate buffered saline) and was then centrifuged at 200,000 × g for 1 hr. All centrifugation steps were done at 4 °C.

### Immunoblotting

Cells were lysed on ice for 30 min, using cold RIPA buffer supplemented with protease inhibitor tablets (Pierce). After centrifuged at 10,000 × g for 30 min at 4 °C, cleared lysates were stored at −80 °C until used for immunoblotting. Samples were run on a 4–12% SDS-PAGE gel prior to transfer to nitrocellulose membranes. Anti-V5 (Invitrogen, Cat# R960-25), anti-Flag (Sigma, Cat#: SAB4200071), anti-acetyl-Lys (Cell Signaling Technology, Cat#: 9441), anti-myc (Covance, Cat# 904401), anti-HA or anti-HA-HRP (Roche, cat#: 11867423001, 12013819001), anti-SIRT1 (Santa Cruz Biotechnology, Cat#: sc-74504), and anti-SIRT2 (Cell Signaling Technology, Cat#: 12650) were used to detect the proteins of interest while anti-actin (Sigma, Cat#: SAB4301137), anti-GAPDH (Millipore, Cat#: AB2302), and anti-tubulin (Abcam, Cat#: AB14128) were used as loading controls. The blots were probed with the indicated primary antibodies followed either by Alexa Fluor-conjugated secondary antibodies for near-infrared fluorescence detection or by HRP-conjugated secondary antibodies for chemiluminescence detection. Unsaturated signals were collected using Hyperfilm ECL (GE healthcare), Odyssey Fc (Li-Cor) or iBright FL1500 (ThermoFisher) imaging system. Full scan of cropped immunoblots were provided in Supplementary Fig. 1.

### Immunoprecipitation (IP)

Cells were washed with ice-cold DPBS (no calcium, no magnesium) and lysed in IP buffer (20 mM sodium phosphate (pH 7.2), 10% glycerol, 250 mM NaCl, 0.1% NP-40, 5 mM EDTA, 400 nM TSA, 5 mM nicotinamide, and protease/phosphatase inhibitors). After centrifugation to clear the insoluble fraction, lysates were incubated with the indicated antibodies overnight followed by incubation with Protein A/G-agarose (Santa Cruz Biotechnology) for 3 hours. After washing agarose beads five times with IP buffer, the captured immunoprecipitates were eluted with x2 Laemmli SDS sample buffer supplemented with a final concentration of 50 mM β-mercaptoethanol.

### Immunofluorescence (IF)

Immunofluorescence was performed as previously described^11^. Briefly, cells were washed twice with DPBS, fixed for 20 min with 4% paraformaldehyde in DPBS, and blocked and permeabilized with 0.7% fish skin gelatin/0.025% saponin in DPBS for at least 3 hrs. DAPI was used to visualize nuclei. GFP-SIRT1, GFP-SIRT2, and GFP as a control plasmid were transfected as described above in order to visualize the distribution of proteins in fixed cells. To stain Lamp1-positive vesicles, anti-LAMP1 (Cell Signaling, Cat#: 9091 S) was used as primary antibody. Confocal images were acquired either Zeiss LSM 780 or Zeiss LSM510.

### CRISPR/Cas9 genome editing

To generate CRISPR/Cas9-mediated gene knock-out lines, Flp-In T-REX HEK293 cells harboring the inducible GPRC5B-HA expression cassette were co-transfected with CRISPR/Cas9 KO sgRNA expression plasmid targeting SIRT1 or SIRT2, together with cognate homology directed repair, HDR plasmids (Santa Cruz Biotechnology, Cat#: sc-40085, sc-40085-HDR, sc-400590, and sc-400590-HDR). Cells with insertional deletion of the indicated target gene through homology-dependent repair were selected with puromycin for 2 weeks, and individual clones were picked for further expansion and chosen when the desired mutation was present.

### Nanoparticle tracking analysis

To measure the sizes and numbers of isolated extracellular vesicles, including exosomes, nanoparticle tracking analysis was performed using the Zetaview (Particle Matrix). Before sample injection, isolated exosome fraction was filtered through 0.2 μm nylon syringe to disaggregate exosomes and all downstream procedures were performed according to the manufacturer’s instruction.

### Statistical analysis

Unpaired Student’s t-test was used to compare the median sizes of particles between control and knockout cells while Kolmogorov-Smirnov test was used to compare particle size distribution. *p-*values < 0.05 were considered statistically significant. Error bars in graphs represent standard error of the mean for three independent experiments. Analyses were performed using Prism 8 (GraphPad).

## Results

### SIRT2 physically interacts with LMAN2

**Figure 1:**
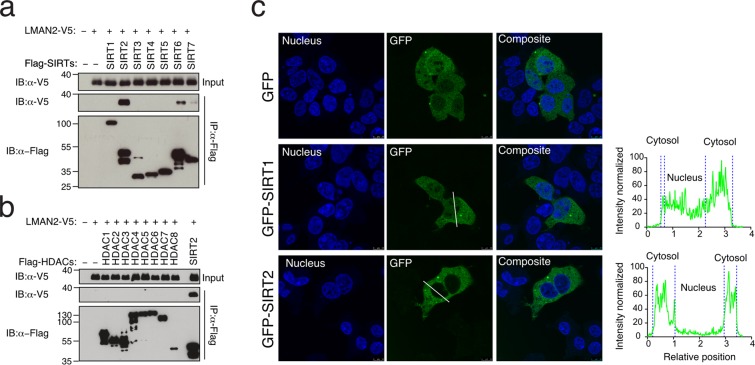
The human deacetylase protein SIRT2 interacts with L-type lectin LMAN2. (**a**) Expression vectors encoding Flag-tagged SIRT 1–7 and V5-tagged LMAN2 were co-transfected with the indicated combinations. Flag-tagged sirtuin (SIRT) proteins were immunoprecipitated with anti-Flag and probed either with anti-Flag or anti-V5. (**b**) Immunoprecipitation was repeated for the histone deacetylase HDAC family of proteins 1–8 using SIRT2 as a positive control. (**c**) Representative confocal images showing GFP, GFP-SIRT1 or GFP-SIRT2 localization. DAPI (blue) is for detection of the nucleus, and GFP (green) for determining the localization of the SIRT proteins in HEK293 cells. Line scan analysis to define SIRT protein distribution. Relative positions are the pixel distances with the first and second maximum pixel intensities measured across the white line indicated in the micrograph. (**c**) Fluorescent signals from DAPI channel was used to define the border of nucleus in the line. Normalized fluorescence intensity through the cell body and nucleus was plotted to quantify the subcellular localization of GFP-tagged proteins.

To understand the mechanism behind LMAN2 functions in exosome biogenesis, we sought to uncover LMAN2 interacting proteins. To accomplish this, we utilized HEK293 cells inducibly expressing GPRC5B-HA that were transiently transfected with an expression plasmid harboring C-terminal V5 epitope tagged LMAN2, LMAN2-V5. After cell lysates were immunoprecipitated with anti-V5, the eluates were analyzed using mass spectrometry to determine any potential binding partners of LMAN2 (data not shown). Although the quality of the data obtained was not ideal, we were able to identify SIRT2 as a potential candidate for an LMAN2 interacting partner. This result seemed plausible because SIRT2 is localized in the cytoplasm of several cell lines^22,23^, and SIRT1, another sirtuin protein has been recently shown to be involved in exosome biogenesis. Since the recent study has been published establishing a role for SIRT1 in exosome release^19,20^, we decided to examine the entire sirtuin protein family for the interaction with LMAN2 and determine if the interaction of SIRT2 and LMAN2 is specific. To do this, we co-expressed LMAN2-V5 protein and each individual sirtuin protein 1 through 7 containing an N-terminal Flag tag in the HEK293 cells (Fig. 1a). When the individual sirtuin proteins were immunoprecipitated as the bait, LMAN2-V5 proteins were detected to a large enough degree in the cell lysates expressing SIRT2 proteins. Although there was also a detectable signal in the cell lysates from SIRT6-transfected cells, the ratio of V5-positive signal to Flag-positive signal was very low, suggesting that while LMAN2 may have the ability to bind to SIRT6, it is likely that there is a stronger preference for SIRT2 interaction towards LMAN2. Additionally, immunostaining showed that SIRT6 was largely localized in the nuclei of HEK293 cells (Supplementary Fig. 2). These observations led us to focus on SIRT2 rather than SIRT6 interaction with LMAN2.

As the most common protein deacetylases in humans fall into two families, namely sirtuin protein deacetylases (SIRTs) and histone deacetylases (HDACs). We also co-transfected individual plasmids encoding Flag-tagged HDAC protein 1 through 8, together with LMAN2-V5 and determined if LMAN2 interacts with HDAC proteins. Immunoblotting analysis (Fig. 1b) showed that none of the HDAC proteins were able to interact with LMAN2 under the same condition. This data further corroborates that the interaction of LMAN2 with SIRT2 is specific. We also performed immunofluorescent staining of SIRT1 and SIRT2 in the HEK293 cells (Fig. 1c). Since LMAN2 is a cytosolic protein involved in exosome biogenesis, we tested if SIRT2 is also found in the cytosol of HEK293 cells when transiently transfected with a plasmid encoding N-terminal GFP-tagged SIRT1 or SIRT2. Indeed, both SIRT1 and SIRT2 proteins were localized predominantly in the cytoplasm of the HEK293 cells. SIRT2 proteins were almost exclusively in the cytoplasm while SIRT1 proteins were present both the nucleus and cytoplasm of the HEK293 cells. Taken together, these results support the physical interaction of LMAN2 with SIRT2 and their potential role in exosome biogenesis and cargo loading. Accordingly, we focused on SIRT1 and SIRT2 to study their roles in exosome biogenesis.

### Overexpressed protein acetyl transferases can lead to increased acetylation of LMAN2

**Figure 2:**
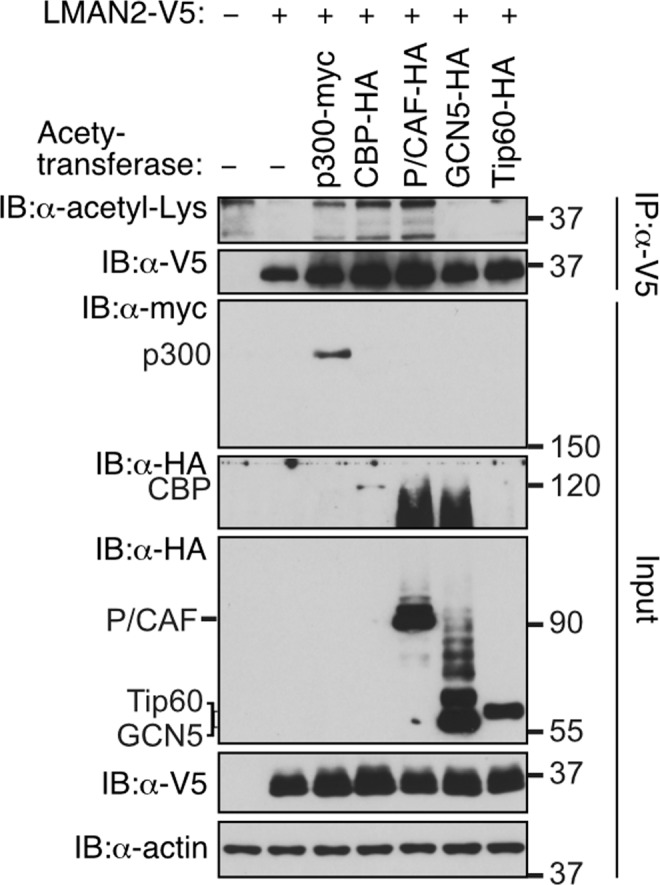
Overexpressed p300, CBP, and P/CAF protein acetyltransferases can lead to LMAN2 acetylation. HEK293 cells were co-transfected with LMAN2-V5 and the indicated acetyl-transferases, p300-myc, CBP-HA, P/CAF-HA, GCN5-HA, or Tip60-HA. LMAN2-V5 proteins were immunoprecipitated with anti-V5 and probed with anti-myc, anti-HA, or anti-acetyl Lys. Acetyl transferase, p300, CBP-HA, and P/CAF increased acetylation of LMAN2, as seen in the immunoblot of acetyl-Lys. The immunoblot for V5 tag was used as an experimental control for the immunoprecipitation and an expression control for the input fraction. The immunoblots for myc and HA were used for visualization of the acetyl-transferase used in the given sample. Actin was used as a loading control for the input fraction.

Next, we determined whether LMAN2 could be acetylated as SIRT2 has protein deacetylase activity. To do this, we co-transfected to HEK293 cells with plasmids encoding LMAN2-V5 along with the indicated protein acetyl transferases (Fig. 2). With the immunoprecipitates using anti-V5 tag, to capture the LMAN2-V5 protein in the lysates, we performed immunoblotting analysis with anti-acetyl-Lys. As shown in Fig. 2, there were detectable but weak acetylation signals only after treatment with p300-myc, CBP-HA, or P/CAF-HA, indicating that these three acetyl transferases might be involved in posttranslational acetylation of LMAN2.

### Partial loss of SIRT2 increases exosome release of GPRC5B

**Figure 3:**
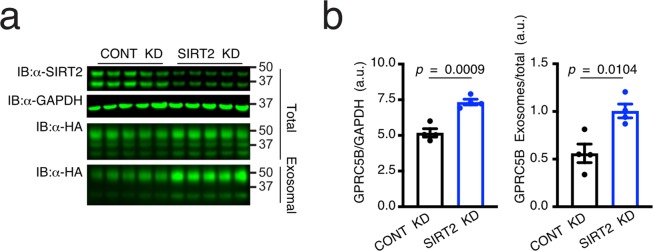
Partial loss of SIRT2 increases exosome release of GPRC5B. (**a**) Immunoblot analyses of cell lysates and exosome fractions from control and SIRT2 knockdown cells for 24-hour doxycycline-mediated GPRC5B induction. GAPDH was used as a loading control. (**b**) Quantitative analyses of the immunoblot signals from nitrocellulose membrane. Note the increased GPRC5B-HA protein in the whole cell lysates and enhanced exosomal release of GPRC5B-HA protein from SIRT2 knockdown cells. Data are shown as mean ± SEM (*error bars*).

Next, to determine if SIRT2 functionally affects cargo loading for exosome release, we transfected shRNAs targeting SIRT2 or negative control shRNAs in the HEK293 cells. Two days after shRNA transfection, GPRC5B-HA expression was induced for 24 hours, as we previously reported^13^. Immunoblotting analysis confirmed partial loss of SIRT2 in cell lysates at three days post shRNA transfection, compared to that in control cell lysates, which was normalized to the expression of GAPDH (Fig. 3a). Knockdown of SIRT2 resulted in a substantial increase of GPRC5B-HA proteins in exosome fraction. Transmission electron microscopy (TEM) analysis of fractionated extracellular vesicles confirmed enriched vesicular entities whose diameters are smaller than 200 nm in the exosome fraction (Supplementary Fig. 3). Partial loss of SIRT2 proteins not only increased cellular expression of GPRC5B but also increased exosome release efficiency of GPRC5B, which is calculated by the ratio of exosome expression to cellular expression of GPRC5B (Fig. 3b). This finding suggests that SIRT2 is a negative regulator for exosomal release of GPRC5B as depletion of SIRT2 increased exosome release efficiency of GPRC5B.

### Loss of SIRT2 results in exosomal release of LMAN2

**Figure 4:**
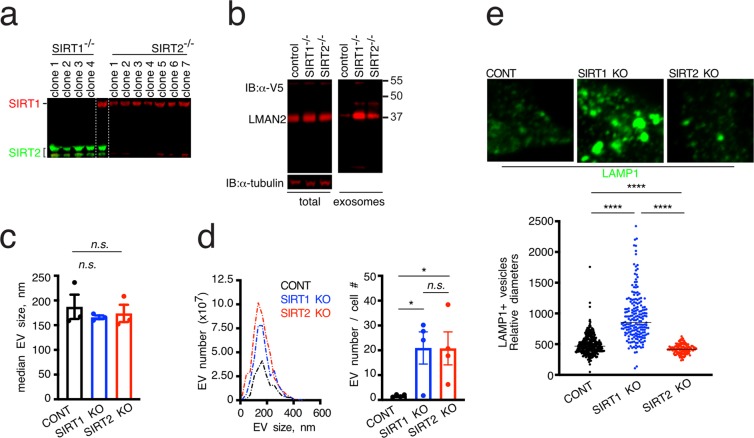
Loss of SIRT2 results in release of LMAN2 via EVs and increased total number of EVs in exosome fraction without endolysosome expansion. (**a**) Immunoblot analysis confirms the loss of SIRT1 or SIRT2 expression in the indicated cell lines generated using CRISPR/Cas9- mediated homology dependent repair. (**b**) Immunoblot analyses of total cell lysates and exosome fractions of control, SIRT1 KO, SIRT2 KO cells. Tubulin was used as a loading control. (**c**) Median sizes of extracellular vesicles (EVs) in exosome fractions. Exosome fraction was prepared as in Materials and Methods section and the size of EVs was determined using nanotracking analysis. Data are shown as mean ± SEM (*error bars*). *n*.*s*., non-significant. (**d**) Total number of EVs and their size distributions were determined. Note that the size distributions of EVs in control, SIRT1 knockout, and SIRT2 knock out were not statistically different when analyzed with Kolmogorov-Smirnov test. Total numbers of exosome released from control, SIRT knockout, and SIRT2 knockout were plotted in the right graph. Data are shown as mean ± SEM (*error bars*). *p < 0.05 (considered statistically significant) as measured by unpaired Student’s *t*-test. (**e**) Loss of SIRT1 expression enlarged LAMP1-positive endolysosomes while loss of SIRT2 expression decreased the size of LAMP-1 positive endolysosomes. Representative immunofluorescence images of fixed cells after anti-LAMP1 labeling (top) was shown along with their quantitation results (bottom). Multiple LAMP1-positive vesicles were randomly selected per cell to determine punta (longest) diameters using ImageJ. Each data point represents an individual LAMP1- positive vesicles and is measured in arbitrary unit. ****p < 0.0001 (considered statistically significant) as measured by unpaired Student’s *t*-test.

To investigate how SIRT1/2 control exosome release of GPRC5B, we generated SIRT1 and SIRT2 knockout (KO) cells lines, using CRISPR/Cas9 technology (Fig. 4a), in which an expression cassette, *pac*, a puromycin-resistant gene was inserted into the *SIRT1* or *SIRT2* gene in the genome via homology-directed repair (HDR) through a Cas9-mediated double strand break. Immunoblotting analysis confirmed inactivation of the target gene by the HDR strategy. SIRT1 or SIRT2 protein expression was not detected in puromycin resistant clones as shown in Fig. 4a. Interestingly, there was no compensation by increased SIRT2 protein levels when SIRT1 was not present and vice versa. To determine if there is a connection between the expression levels of LMAN2 and SIRT1/2, knockout cells were transfected with LMAN2-V5, and then cell lysates and exosomes which were collected from the conditioned media exposed to the cells for 24 hours were subjected to immunoblotting with anti-V5 (Fig. 4b). Based on our previous findings, we expected that the levels of cellular LMAN2 decrease in the SIRT2 knockout line, thereby leading to increased GPRC5B in exosomes fraction. However, there was no significant difference in the expression levels of LMAN2-V5 in the lysate from the knockout cells compared with that from control cells. Unexpectedly, however, we found a large increase of LMAN2-V5 in the exosome fractions from both SIRT1 and SIRT2 knockout cells. Taken together, these results suggest that acetylation of LMAN2 or other acetylated proteins enhances exosome cargo release. Alternatively, the protein interaction of LMAN2 and SIRT2 is required for intracellular retention of LMAN2, which in turn limits exosomal release of GPRC5B.

### While loss of SIRT1 increases total number of extracellular vesicles with endolysome enlargement, loss of SIRT2 increases release of EVs without the enlargement

To test the presence of detectable differences to exosome biogenesis in SIRT1 and SIRT2 knockout cell lines, we next examined both the size and number of released extracellular vesicles (EVs) over 22 hours (Fig. 4c,d). While there were no significant differences in the median sizes and overall size distributions of EVs (Fig. 4c), there were highly significant changes in the total number of EVs in exosome fractions collected in each cell lines (Fig. 4d). Both SIRT1 and SIRT2 knockout cells released significantly more EVs than the control cells did, suggesting that both SIRT1 and SIRT2 are implicated in EV biogenesis by increasing the flux to vesicle release. Finally, we determined if loss of SIRT2 expression has similar endolysosomal irregularities that have been reported in loss of SIRT1 expression^19^. To do this, we fixed the cells and performed immunofluorescence staining with anti-LAMP1 to visualize endolysosomal structures (Fig. 4e). While the SIRT1 knockout cells contained significantly larger LAMP1-positive endolysosomes that replicated as in the previously published finding, SIRT2 knockout cells have slightly but significantly decreased endolysosome sizes. As shown in Supplementary Fig. 4, knockout of SIRT1 and SIRT2 did not affect cell size.

## Discussion

As a follow-up to our previous publication which found that the protein LMAN2 is involved in cargo loading and transport along the exosomal pathway^13^, we sought to identify novel interactors to LMAN2. We found that SIRT2 has a specific interaction with LMAN2 that is not seen with other human protein deacetylases. We also determined that SIRT2 is a negative regulator of exosome biogenesis as noted by an increased GPRC5B in exosome fractions when SIRT2 was depleted. This is interesting because we previously reported that LMAN2 is also a negative regulator of GPRC5B cargo loading in exosome biogenesis. Although it remains undetermined if the interaction between LMAN2 and SIRT2 is direct or indirect, our current study indicates that the physical interaction is likely a mechanism for controlling exosome release of GPRC5B. LMAN2 has been shown to regulate exosomal release of GPRC5B at the Golgi by limiting traffic from the Golgi to MVB^13^. Since depletion of SIRT2 allows LMAN2 to exit from the Golgi, LMAN2 is no longer able to limit GPRC5B from the exosome release traffic route. Our data also suggest that post-translational protein acetylation might control cargo loading for exosome release as well as the number of exosomes released into extracellular milieu.

While conducting our experiments to study the role of sirtuin proteins in exosome biogenesis, Latifkar and colleagues reported the role of SIRT1 in exosome release from a breast cancer cell line^19^. In their study, knockdown of SIRT1 resulted in a decrease of V-ATPase, an enzyme that is required for lysosomal protein degradation. Reduced SIRT1 expression caused larger MVBs, which contained a larger amount of cargo that was often ubiquitinated and were less readily trafficked to the lysosome. Despite having lower numbers of MVBs interacting with the lysosome, this subcellular structure was significantly enlarged leading to an increase in exosome biogenesis. Ultimately, it was concluded that partial loss of SIRT1 reduces V-ATPase expression and inhibits the lysosome, which prevents MVB degradation, thereby promoting the release of exosome cargo. These findings complement other publications stating that exosome biogenesis can be impaired by altered lysosomal function via aberrant MVB formation and fusion^24,25^. Consistent with this finding, loss of SIRT1 in our experimental setting resulted in increased release of EVs which was accompanied with increased LAMP1-positive late endolysome sizes.

Our study expands further on this recent finding by linking LMAN2 to the presence of SIRT2 proteins as a new player in exosome biogenesis and furthermore providing a different mechanism of SIRT2 and SIRT1 in exosome biogenesis. Increased exosomal release of LMAN2 (and in turn GPRC5B) implies that this is a specific pathway, representing cargo loading/traffic for GPRC5B. Does SIRT2 act only on GPRC5B or on other cargo proteins in exosomes and extracellular vesicles in general? The latter would suggest that SIRT2 might also affect the amount of entire populations of exosome and EV proteins that transit the endocytic pathway. To address this, we measured total number of EVs released in SIRT2 knockout cells. As observed in SIRT1 knockout cells, total EV numbers were remarkably increased in SIRT2 knockout cells. Next, we asked whether SIRT2 acts like SIRT1 by impairing the lysosomal pathway. Strikingly, the increased EV release observed in SIRT2 was not associated with enlarged endolysosomes as in SIRT1 knockout cells, suggesting there is a distinct mechanism by which SIRT2 increases EV biogenesis though the precise mechanism is still not known. In sum, our data favor the following two models where LMAN2 is regulated by direct or indirect posttranslational acetylation that controls cargo loading destined to EVs. Alternatively, the physical interaction of LMAN2 proteins with SIRT2 proteins is required for intracellular retention of exosome cargo, including GPRC5B, thereby suppressing exosome release. Both models indicate SIRT2 controls cargo loading of exosome proteins. In addition to the cargo loading, SIRT2 can increase EV (and exosome) flux as observed in SIRT1 but the underlying mechanisms are different between these two sirtuin proteins.

The results from these experiments provide novel information on the role of sirtuin and LMAN2 on the EV (and exosome) cargo pathway. This information combined with prior evidence suggesting that sirtuin (SIRT) proteins are potential therapeutic targets for kidney disease provides a rationale for further studies examining the integration between sirtuin and LMAN2. Since sirtuin proteins can modulate exosome biogenesis and are protective against kidney injury^26,27^, it becomes a point of interest to determine whether sirtuin proteins can reflect injured kidney status in EVs. Ultimately, this information would be translational and could potentially lead to novel therapeutic intervention strategies.

## Supplementary information

Supplementary Information
