# Supplementary material for: Distinct role of Sirtuin 1 (SIRT1) and Sirtuin 2 (SIRT2) in inhibiting cargo-loading and release of extracellular vesicles
Source: Sci Rep. 2019 Dec 27;9:20049. doi: 10.1038/s41598-019-56635-0 (PMC6934595; doi:10.1038/s41598-019-56635-0)
Supplement: Supplementary file 1 — Supplementary Information [file 41598_2019_56635_MOESM1_ESM.pdf]

Supplementary Information

Distinct role of Sirtuin 1 (SIRT1) and Sirtuin 2 (SIRT2) in inhibiting cargo-loading and release of extracellular vesicles

Byung Rho Lee, Bethany J. Sanstrum, Yutao Liu, and Sang-Ho Kwon*

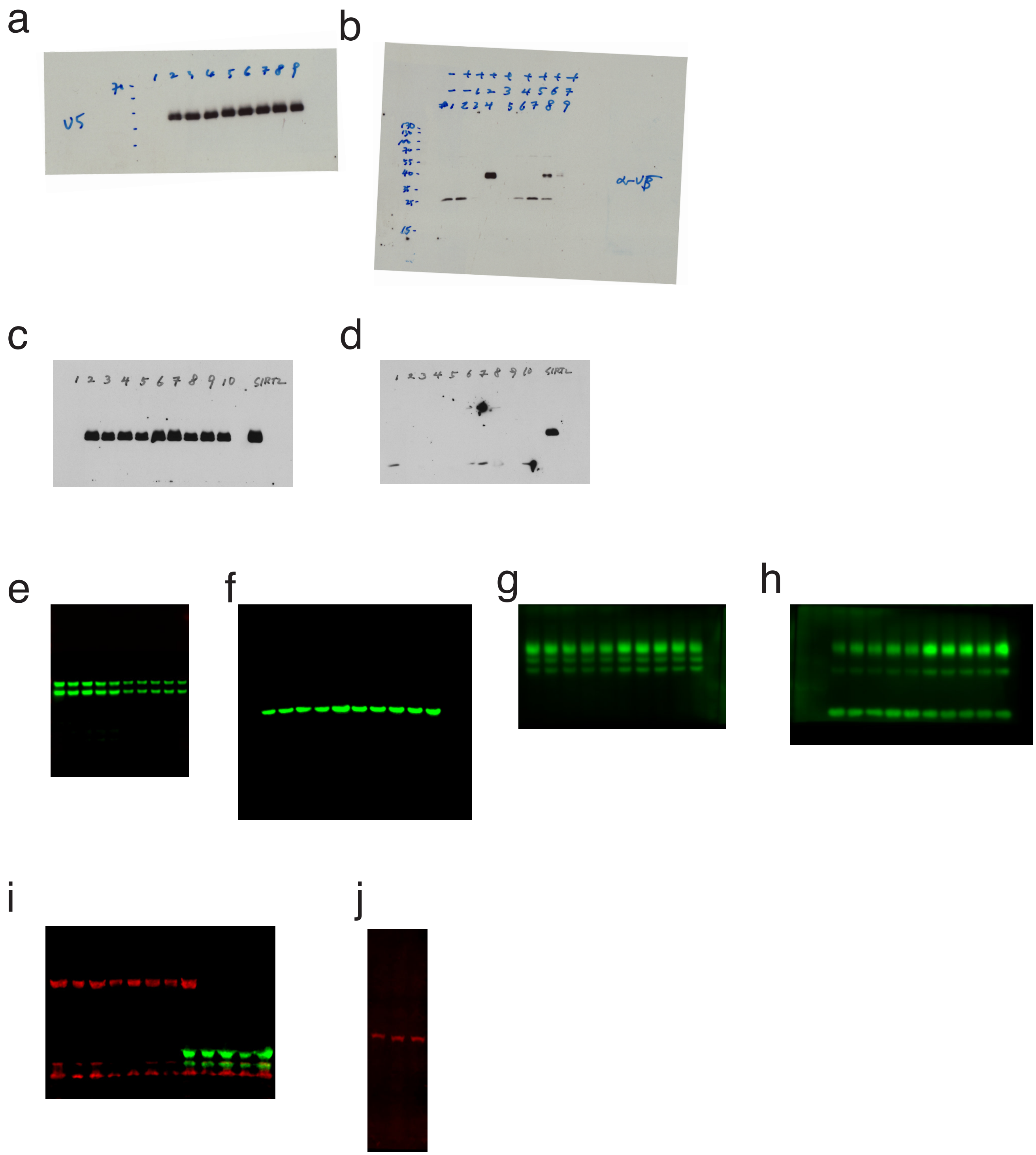

Supplementary Figure 1. Full scan of cropped Immunoblots. a) Fig.1a, Input IB: anti-V5, b) Fig.1a, IP: anti-Flag IB: anti-V5 c) Fig.1b Input IB: anti-V5, d) Fig 1b, IP: anti-Flag IB: anti-V5, e) Fig. 3a, Total IB: anti-SIRT2, f) Fig. 3a, Total IB: anti-GAPDH g) Fig. 3a, Total IB: anti-HA, h) Fig. 3a, Exosomal IB: anti-HA, i) Fig. 4a, IB: anti-SIRT1 and anti-SIRT2, j) Fig. 4b, Total IB:anti-tubulin

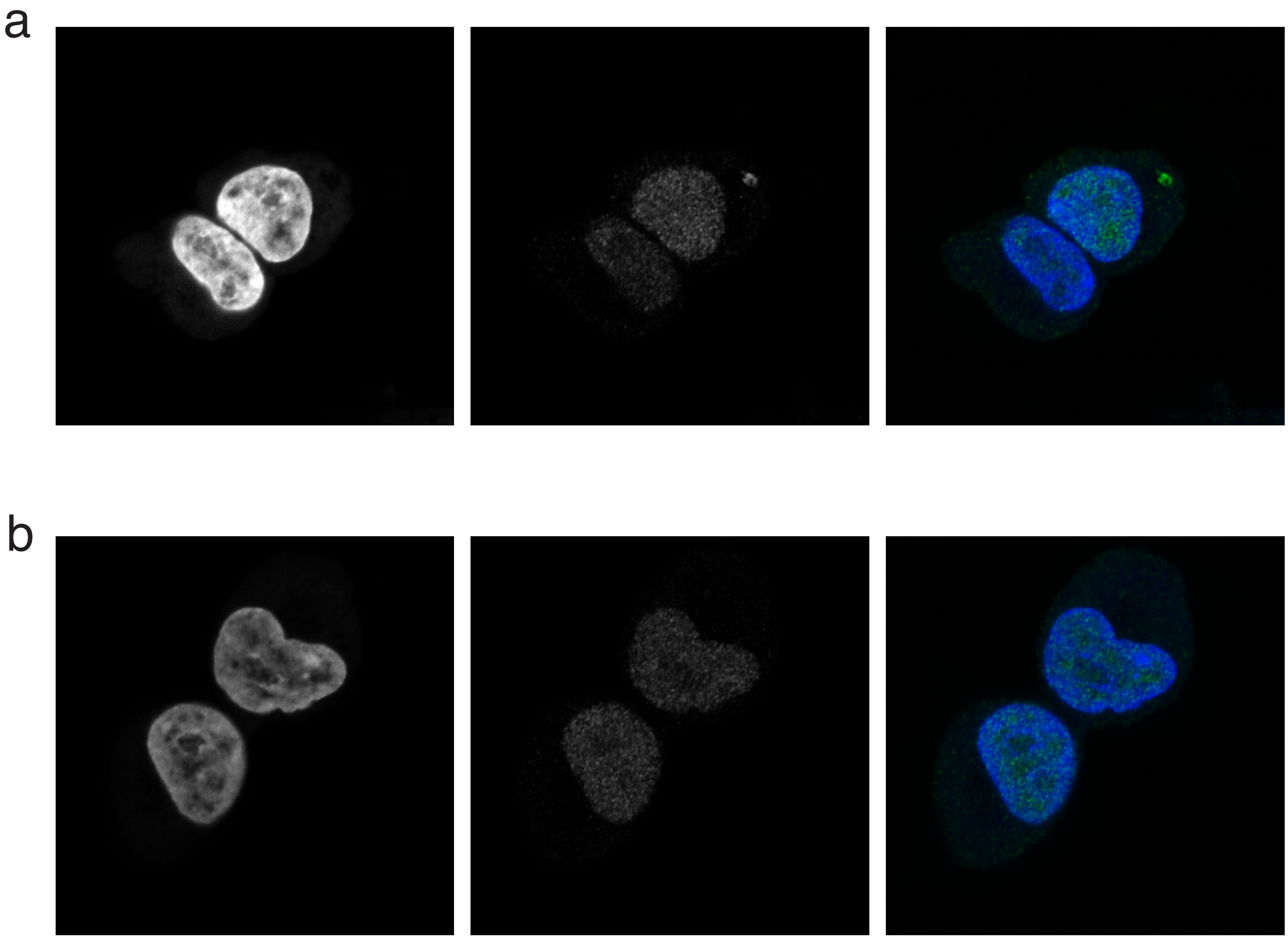

Supplementary Figure 2. Representative confocal images of nuclear localization of SIRT6.
DAPI (blue) is for detection of the nucleus and SIRT6 (green) for determining localization of SIRT6 in HEK293 cells. Note that both anti-SIRT6, ab62739 from Abcam used in (a) and 12486S from Cell Signaling Technology used in (b) indicated nuclear localization of SIRT6 in HEK293 cells.

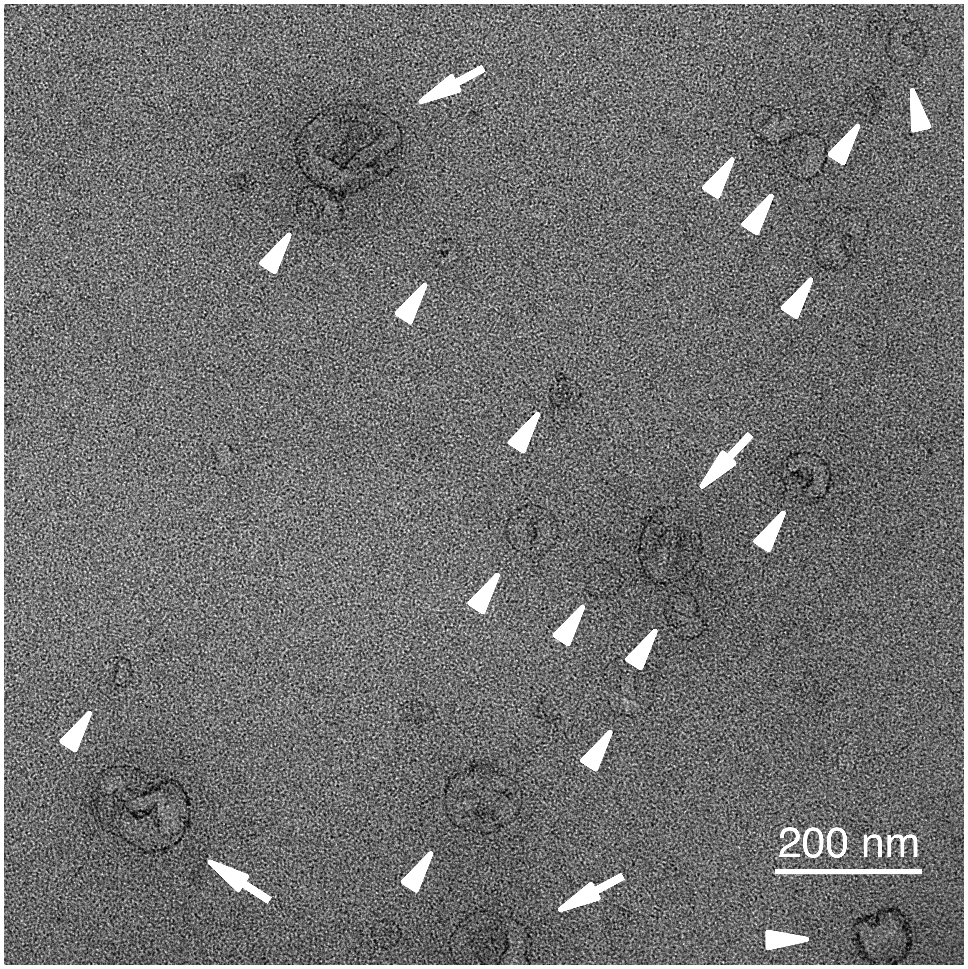

Supplementary Figure 3. Transmission electron microscopy image of the exosome fraction used in this study. Resuspended exosome-containing pellet was fixed on electron-microscope grids, and contrasted and embedded to visualize vesicles in the fraction. Arrowheads indicate extracellular vesicles with the diameter < 100 nm while arrows indicate vesicles with 100 - 200 nm diameter. Note vesicles < 200 nm were enriched in the exosome.

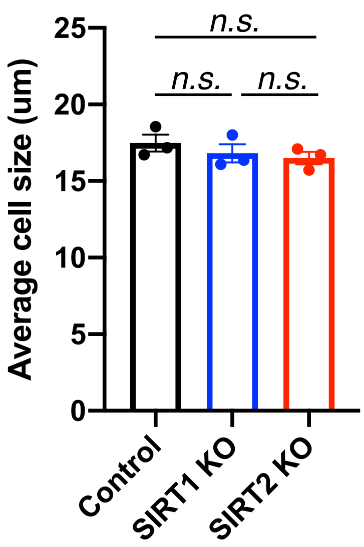

Supplementary Figure 4. Loss of SIRT1 or SIRT2 did not affect cell size. Average cell sizes from live single cell resuspension of 2.89 x10^6 of control, 5.47 x10^6 of SIRT1 KO, and 4.78 x 10^6 of SIRT2 KO cells were measured using Countess II FL. Data are shown as mean ± SEM (*error bars*) n.s., non-significant. Data points obtained from three independent experiments.
